# Orientation of native versus translocated juvenile lesser spotted eagles (*Clanga pomarina*) on the first autumn migration

**DOI:** 10.1242/jeb.148932

**Published:** 2017-08-01

**Authors:** Bernd-U. Meyburg, Ugis Bergmanis, Torsten Langgemach, Kai Graszynski, Arno Hinz, Ingo Börner, Christiane Meyburg, Wouter M. G. Vansteelant

**Affiliations:** 1 BirdLife Germany (NABU), PO Box 330451, Berlin 14199, Germany; 2 Latvijas valsts meži, Vaiņodes iela 1, Rīga LV –1004, Latvia; 3 Brandenburg State Bird Conservation Centre, Dorfstr. 34, Buckow, Nennhausen 14715, Germany; 4 Department of Biology, Free University Berlin, Schreberstr. 8 A, Berlin 14167, Germany; 5 Agency of Forestry, Vietmannsdorfer Str. 39, Templin 17269, Germany; 6 Veterinarian practice, Neuer Weg 5, Templin 17268, Germany; 7 World Working Group on Birds of Prey, 31, Avenue du Maine, Paris 75015, France; 8 Theoretical and Computational Ecology, Inst. for Biodiversity and Ecosystem Dynamics, University of Amsterdam, Amsterdam, The Netherlands; 9 Vansteelant Eco Research, Dijkgraaf 35, Bennekom 6721NJ, The Netherlands

**Keywords:** Bird migration, Orientation, Satellite-tracking, Translocation

## Abstract

The ontogeny of migration routines used by wild birds remains unresolved. Here we investigated the migratory orientation of juvenile lesser spotted eagles (LSE; *Clanga pomarina*) based on translocation and satellite tracking. Between 2004 and 2016, 85 second-hatched juveniles (Abels) were reared in captivity for release into the declining German population, including 50 birds that were translocated 940 km from Latvia. In 2009, we tracked 12 translocated juveniles, as well as eight native juveniles and nine native adults, to determine how inexperienced birds come to use strategic migration routes. Native juveniles departed around the same time as the adults and six of eight used the eastern flyway around the Mediterranean, which was used by all adults. In contrast, translocated juveniles departed on average 6 days before native LSEs, and five travelled southward and died in the central Mediterranean region. Consequently, fewer translocated juveniles (4/12) than native juveniles (7/8) reached Africa. We conclude that juvenile LSEs have a much better chance of learning the strategic southeastern flyway if they leave at an appropriate time to connect with experienced elders upon departure. It is not clear why translocated juveniles departed so early. Regardless, by the end of the year, most juveniles had perished, whether they were translocated (10/12) or not (6/8). The small number of surviving translocated juveniles thus still represents a significant increase in the annual productivity of the German LSE population in 2009.

## Abstract (precis)

**Highlighted Article:** In 2009, most juvenile eagles translocated from Latvia to Germany departed earlier than native juveniles and adults, and consequently failed to learn the species' traditional eastern detour around the Mediterranean.

## INTRODUCTION

The advent of satellite telemetry and GPS tracking sparked a new age of discovery in animal ecology (Kays et al., 2015). This is especially true for the study of bird migration (Bridge et al., 2011; Robinson et al., 2010; Wikelski et al., 2007). Tracking entire cross-continental or oceanic journeys of migrant birds has revealed that migrants often adhere to highly complex migration itineraries, which may differ greatly between individuals from the same species or population (Åkesson and Hedenström, 2007; Thorup et al., 2010; Vardanis et al., 2016). Complex migration routines, sometimes requiring accurate navigation toward crucial stop-over and staging sites, may be explained to some extent by relatively simple, genetically determined migration programs that involve one or more bouts of vector-based navigation (Berthold and Terrill, 1991; Mouritsen and Mouritsen, 2000), and some simple behavioural rules, such as aversion to crossing open water and other barriers (Erni et al., 2003; Hake et al., 2003; Kerlinger, 1985; Meyer et al., 2000; Thorup et al., 2003). Such simple mechanisms may be particularly relevant for nocturnally migrating passerines and other solitary migrants. For other migrants, however, including large diurnal migrants such as soaring birds, social learning is likely to play a much more important role. Species such as cranes (*Grus* sp.), white stork (*Ciconia ciconia*), Egyptian vulture (*Neophron percnopterus*), lesser spotted eagle (LSE; *Clanga pomarina*), short-toed eagle (*Circaetus gallicus*) and European honey buzzard (*Pernis apivorus*) all depend on guidance by elders to locate strategic flyways or important stop-over sites (Chernetsov et al., 2004; Hake et al., 2003; Mellone et al., 2011; Meyburg et al., 2016; Rotics et al., 2016; Schüz, 1950). It is likely that most birds use a combination of innate vector-based (or more complex) orientation programs and social information, as well as multiple additional cues to facilitate accurate navigation early in life (Åkesson and Hedenström, 2007; Thorup et al., 2010; Wiltschko and Wiltschko, 2015). As Thorup et al. (2010) argued: ‘how else could one account for the complex routes, such as those used by juvenile Eleonora's falcons *Falco eleonorae* travelling to a restricted wintering range in Madagascar, in the absence of experienced elders (Gschweng et al., 2008)?’ However, besides cranes and geese, most social migrants do not travel in family groups, and so it remains unclear how long after dispersal it takes for naive juveniles to encounter elder conspecifics, what they do in the absence of elder guides and how social learning affects their survival.

Outside of the laboratory, the best way to empirically study the role of innate versus external influences on migratory behaviour is through displacement or delayed-release experiments (Perdeck, 1958; Schüz, 1950). Few migratory behaviour studies of displaced birds have been performed using tracking devices (Chernetsov et al., 2004; Thorup et al., 2010; Wikelski et al., 2015; Willemoes et al., 2015). Moreover, very few tracking studies have been conducted on juvenile migrants because naturally higher juvenile mortality rates mean that young birds are likely to die shortly after they are fitted with an expensive transmitter, and also because transmitters may affect juveniles relatively more than adults. So far, juvenile tracking studies have mostly been conducted on large soaring birds, and although in some species juveniles travel faster than adults (Rus et al., 2017), juveniles from wild populations are usually outperformed by adults (Rotics et al., 2016; Sergio et al., 2014), migratory performance improves with age (Sergio et al., 2014) and juveniles are often guided by elders to migrate (Mueller et al., 2013; Oppel et al., 2015; Rotics et al., 2016; Sergio et al., 2014). In the absence of displacement or delayed-release experiments on juvenile birds, however, it is impossible to disentangle the relative influence of innate behaviours from social information in the development of migratory repertoires in individual birds from natural populations (Muriel et al., 2016; Senner et al., 2015). This knowledge gap must be addressed to develop a comprehensive ontogenetic perspective on migratory orientation (Guilford et al., 2011; Senner et al., 2015; Thorup et al., 2010) and concomitant patterns of migratory connectivity (Cresswell, 2014; Trierweiler et al., 2014), in addition to determining how migration affects population numbers (Hewson et al., 2016; Newton, 2006). A better understanding of migratory development could also help to identify conservation priorities in the annual cycle and life cycle of migrant birds (Hostetler et al., 2015; Vickery et al., 2014).

The present study aimed to determine how translocation affects the route choice of juvenile LSEs (*Clanga pomarina* Brehm 1831) (Lerner et al., 2017) during the first autumn migration*.* In 2009, 20 solar-powered Argos GPS platform transmitter terminals (PTTs, 22 g) were deployed on juvenile eagles. Twelve of these juveniles were second-hatched juveniles from Latvia that were reared in captivity (hereafter ‘hacked’, see Materials and methods) and translocated approximately 940 km southwestward to supplement the declining population in Brandenburg, Germany. The other eight juveniles were reared in the wild in northeastern Germany. Moreover, of 48 adult LSEs that were tracked since 1994 (Meyburg et al., 1995, 2004a), 10 adults that bred in Germany were still transmitting in 2009. Consequently, for that year, we were able to compare the migration timing and route choice of translocated juveniles, native juveniles and native adults. The eagles were tracked primarily to monitor the effectiveness of hacking and translocation as a conservation measure (Graszynski et al., 2011; Meyburg et al., 2008). Our focus here, however, is to derive fundamental insights from this quasi-experimental study into the factors driving decision-making in a juvenile soaring migrant.

Being among the largest Palearctic soaring migrants, LSEs are especially vulnerable to exhaustion when forced to travel by flapping flight, such as during long overwater flights (Bildstein et al., 2009; Oppel et al., 2015). Consequently, most of the LSE population avoids sea crossings, and migrates over land by thermal soaring (Kerlinger, 1985; Meyburg et al., 2002). Most LSEs that make successful autumn migrations travel along the eastern Mediterranean flyway, from the Bosphorus to Suez and along the Great Rift Valley into central and southern Africa (Bijlsma, 1983; Leshem and YomTov, 1996; Meyburg et al., 1995, 2008; Michev et al., 2011). The use of this eastern migration route is a highly conserved trait in the migration of the LSE, as evidenced by the fact that all adult LSEs that have ever been tracked for more than 1 year (48 individuals), including individuals that were tracked for up to 11 consecutive years (Meyburg et al., 2004a, 2015), used the eastern route every autumn and spring. A small number of adult and juvenile LSEs do migrate along southwestern and southern flyways via the Strait of Gibraltar (Onrubia et al., 2011) or the Strait of Sicily between Italy and Tunisia (Thiollay, 1975; Dejonghe, 1980; Giordano, 1991).

Some juvenile migrant birds are able to compensate for extremely large displacements resulting from either natural causes, such as extreme weather, or unnatural causes, such as experimental interventions (Thorup et al., 2011). True goal navigation, however, is far more common among adult migrants (Perdeck, 1958), and even such experienced individuals may elicit highly variable responses to displacement (Willemoes et al., 2015), in some cases failing to compensate at all (Kishkinev et al., 2016). Therefore, we did not expect translocated juveniles to compensate for their displacement either by returning to their natal site or by compensatory goal navigation towards the southeastern flyway. Instead, we expected that both translocated and native juveniles would locate the southeastern flyway using social information (Chernetsov et al., 2004; Schüz, 1950). Because Germany is situated at the western limit of the species' range, and because very few adults breeding to the north and further east pass through this peripheral population on migration, native adults (and non-breeding immatures) should be the main providers of social information for juveniles departing from our study population (Oppel et al., 2015). Therefore, we expected that both translocated and native juveniles would be more likely to learn the eastern route if they departed simultaneously with native elders.

Juveniles that fail to synchronize their departure with elders likely rely on innate, vector-based orientation to find their way to sub-Saharan Africa (Thorup et al., 2003). Although LSEs frequently migrate in mixed-age groups, they do not seem to migrate in family groups (Meyburg and Meyburg, 2007; B.-U.M. and C.M., unpublished data); family group migration is also not a characteristic of other raptor species such as the closely related greater spotted eagle (*Clanga clanga*; Meyburg et al., 2005). Consequently, to maximize the opportunities to learn the route via the Bosphorus and Suez, the juveniles should time their departure to coincide with the departure or passage of adult LSEs, and their innate departure directions should be oriented from the natal area toward the southeastern flyway. We did not expect a difference in timing of departure between native and translocated juvenile LSEs, but we predicted that if translocated and native juveniles departed much earlier or later than adults, they would default to southward and eastward–southeastward departure directions, respectively. As a consequence, translocated juveniles would likely end up in the central Mediterranean flyway if they failed to connect with native adults upon departure (Meyburg et al., 1995, 2008). Given that heavy soaring birds often perish when they attempt long sea crossings (Bildstein et al., 2009; Mellone et al., 2011; Oppel et al., 2015), the failure of translocated juveniles to locate and follow elder guides using the southeastern flyway could constrain the effectiveness of translocation and hacking in reinforcing the declining LSE populations in Germany and elsewhere.

## MATERIALS AND METHODS

### Lesser spotted eagles in Germany

The LSE is a large soaring raptor and long-distance migrant that breeds in moist woodlands from central Europe to western Russia (Ferguson-Lees and Christie, 2006; Meyburg et al., 2001, 2016). Since 1992, we have tracked 95 LSEs via satellite. The large majority of tracked LSEs leave Europe in September, and all adults use the eastern Mediterranean flyway via the Bosphorus and Suez en route to Africa. A few thousand adults and immatures breeding in the eastern part of the range migrate along the eastern Black Sea flyway. These two routes converge over Turkey and Syria as LSEs travel toward Suez. Few LSEs cross the Mediterranean through the Straits of Gibraltar and via Sicily (Agostini et al., 2004; Onrubia et al., 2011; Giordano, 1991); these flyways are clearly of minor importance compared with the southeastern flyway.

### Saving Abel: nestling management for conservation

The western limit of the species' breeding range lies in Germany, where a small population persists despite a major population decline from the 1990s to the 2000s (133 pairs in 1993 to 107 pairs in 2007, 23% decline; Meyburg et al., 2004b, 2008). Since 2001, the breeding population of LSE in the main German stronghold in Mecklenburg-Vorpommern has declined from 92 to 87 breeding pairs (LUNG, 2016). Because the LSE is red-listed in Germany (Grüneberg et al., 2015; Vökler et al., 2014), an action plan was developed to conserve it nationally, including habitat protection measures complemented by a population recovery program (Meyburg et al., 2004b; MLUV, 2005).

LSEs typically lay two eggs, both of which usually hatch. Under natural conditions, however, the second-hatched juvenile is killed by the first-hatched, a process referred to as obligate siblicide or ‘Cainism’ (after the biblical Cain and Abel struggle; Meyburg, 1974, 2002). Removing the second-hatched chicks before they are killed, rearing them in captivity and then releasing them can be a way of supplementing native productivity, and may help to halt population declines (Meyburg, 1978). Such a program of conservation-focused nest management has been implemented by the ‘Saving Abel’ project in Germany since 2004 (Meyburg et al., 2008; Graszynski et al., 2011), and between 2004 and 2016, 85 Abels were released in the German state of Brandenburg (Graszynski et al., 2011; B.-U.M. and C.M., unpublished data). This intervention increased the number of birds that fledged from the Brandenburg population by 55.9%, during a time period when only 144 juveniles fledged in the wild. Of these 85 birds, 35 individuals were taken from eyries in Brandenburg, and 50 were translocated from eyries in the core breeding range of LSE in Latvia, some 940 km to the northeast. Most Abels were sourced from populations in Latvia owing to the limited size of the Brandenburg population (ca. 20 pairs; Langgemach et al., 2010).

### Translocating and releasing juvenile eagles

The Abels taken from Latvian eyries were initially reared near their natal sites, then transferred to the Riga Zoo until they reached approximately 5 weeks of age, and then flown to Berlin and taken to a release station (53°N, 13°30′ E) in the UNESCO-biosphere reserve of Schorfheide-Chorin in Brandenburg. Until 2008, the Abels were captive-reared until they were old and fit enough to compete for food with a nest mate, then fostered to wild breeding parents. In 2009, so as to avoid potential problems with food provisioning and losses owing to predation, the fostering method of releasing birds was replaced by hacking (Sherrod, 1982; Graszynski et al., 2011; Meyburg et al., 2008). The hacking station was situated within the core breeding area of LSE in Brandenburg and within 100 km of all known nests. Release of hacked juveniles took place in late July to mid-August, 5 to 6 weeks before the onset of autumn migration. The young eagles moved around freely after release, and the vast majority remained in the vicinity of the hacking station, where plentiful food is provided, until they start to migrate (Graszynski et al., 2011; Meyburg et al., 2008).

Hatching date, date of placement in the hacking station, release date and date of departure from the hacking station were recorded for all translocated juveniles (Table S1). For comparison, we recorded fledging dates of wild juveniles in Latvia as determined from nest camera footage (*n*=4). Both translocated and native juveniles were fully grown with fully developed wings at the time that translocated juveniles were released from the hacking station, indicating no delay or advance in physical development of translocated, hacked juveniles versus native juveniles.

### Tracking juvenile and adult eagles

We tracked 12 translocated juveniles (Abels) and eight native (wild) juveniles (Cains) in 2009 as they migrated from Germany (Table 1). Deployment of the transmitters took place approximately 1 week before fledging, for both the native and the translocated eagles (Table S1). To understand juvenile behaviour in the context of elder migration strategies, we used tracking data from 10 adult LSEs with active PTTs in 2009 (Table 1), seven of which had already been tracked for several years and three of which were trapped and marked in 2009. For comparison, we included satellite-tracking data of a non-translocated juvenile that fledged from an eyrie in Latvia in 1993 and an adult breeding in Latvia in 1997.

**Table 1.**
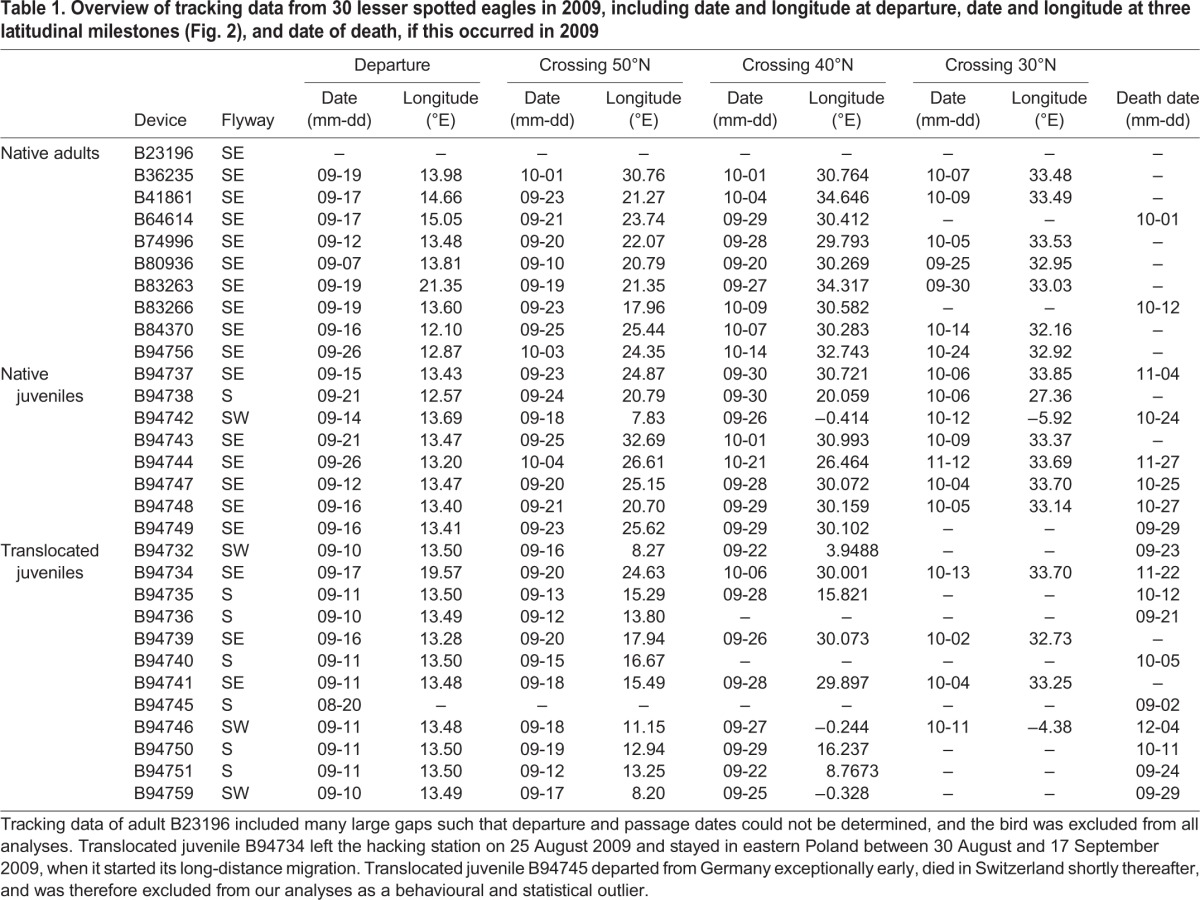
**Overview of tracking data from 30 lesser spotted eagles in 2009, including date and longitude at departure, date and longitude at three latitudinal milestones (****Fig. 2****), and date of death, if this occurred in 2009**

For each bird, we determined the date of departure as accurately as possible based on GPS fixes, complemented by Argos/Doppler fixes (LC 3,2,1, as well as lower quality fixes with coordinates at a bird's nest site or at the hacking station) when there were gaps in the GPS data. Visual examination of the tracks revealed that all birds used one of the previously described flyways to Africa: the southwestern flyway (via Gibraltar), the southern/central flyway (via the central Mediterranean) or the southeastern flyway (via the Bosphorus and Israel). We recorded the longitude and date of passage at three latitudinal thresholds, chosen to reflect three distinct milestones in the first outbound migration: successful departure, completion of migration through Europe, and arrival in Africa after crossing or circumventing the Mediterranean Sea. Finally, based on expert interpretation of the GPS and Argos data, we determined the tracks that ended owing to mortality before 31 December 2009. The causes of mortality of these and other tracked young and adult birds will be discussed in another paper. Here, mortality was confirmed in the field or assumed to be highly likely when tracks ended just before or during crossing of the sea or the Sahara, at known hot-spots for illegal shooting of migrant birds, or if a bird remained stationary for a long period of time before contact was lost.

### Analyses

Adult LSEs travelled along highly similar routes, with all individuals using the southeastern flyway. Our main interest was to determine how the origin and timing of migration by juveniles affects the extent to which their migration behaviour diverges from that of the native adults. Thus, we determined the extent to which birds using each of the three flyways differed between translocated juveniles and native eagles using a Fisher's exact test (Freeman and Halton, 1951). We ran ANOVAs to determine whether the date and longitude at departure and at latitudinal milestones differed between translocated and native juveniles, between native juveniles and adults, and between translocated juveniles and native adults, while adjusting for the chosen flyway. Using a log-rank test, we compared survival between groups and between flyways.

### The influence of topography and wind conditions at departure

Differences in local topography between departure locations and in weather conditions between the days that eagles departed may drive part of the observed variation in route choice. Therefore, we mapped the routes of all LSEs relative to the topography of the surrounding landscape at departure. In addition, we produced detailed maps to explore the influence of topography on route choice over eastern Europe and eastern Africa.

To detect confounding effects of westward and eastward winds on the longitudinal displacement at departure, we annotated all tracks of birds departing from Germany with the westward/eastward wind component from the NCEP-NCAR reanalysis model (Kalnay et al., 1996; Kemp et al., 2012). To do this, we linearly interpolated u-wind components from the 925 mB pressure level (corresponding to an average flight altitude of approximately 700 m) across the 2.5×2.5° NCEP grid to each GPS fix obtained north of latitude 50°N. We then ran generalized linear regression models to determine the relative effect of age, geographic origin (i.e. native versus displaced), and the mean amount of westward/eastward wind experienced north of latitude 50°N on the longitude at which birds crossed latitude 50°N. Next, we separately tested for the influence of westward/eastward side winds on the longitudinal displacement at departure for each group of eagles. In addition, we investigated whether selection of wind conditions could have affected departure timing by plotting departures on a graph of daily wind conditions at 12:00 UTC during September 2009 in eastern Germany (53.5°N, 13.5°E).

**Fig. 1.**
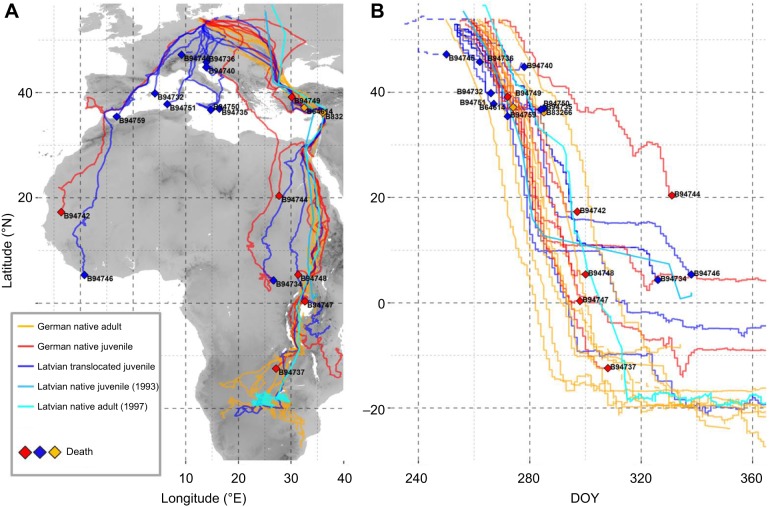
**Routes and timing of migration by 12 juvenile lesser spotted eagles (LSEs) translocated from Latvia to Germany (dark blue) compared with eight native juvenile LSEs (red) and 10 native adult LSEs (orange) in autumn 2009.** In addition, we mapped and graphed older tracking data (light blue) of a native Latvian juvenile tracked in 1993 and a native Latvian adult tracked in 1997 (bright blue). (A) The map shows migration routes; labelled diamonds indicate locations where birds died. (B) Migration timing is shown as the latitude of each GPS fix in relation to the day of the year (DOY; 1–365, whereby 250=7 September and 300=27 October); mortality events are indicated by labelled diamonds. Dashed tracks indicate exceptionally early movements by B94734 and B94745 that were not included in our analyses because they were deemed to be behavioural and statistical outliers. Tracks that terminate before the end of the year but are not labelled with a diamond (e.g. Latvian native juvenile in 1993 and some native German adults in 2009) are from birds that survived, but for which a gap in data occurred at year end.

**Fig. 2.**
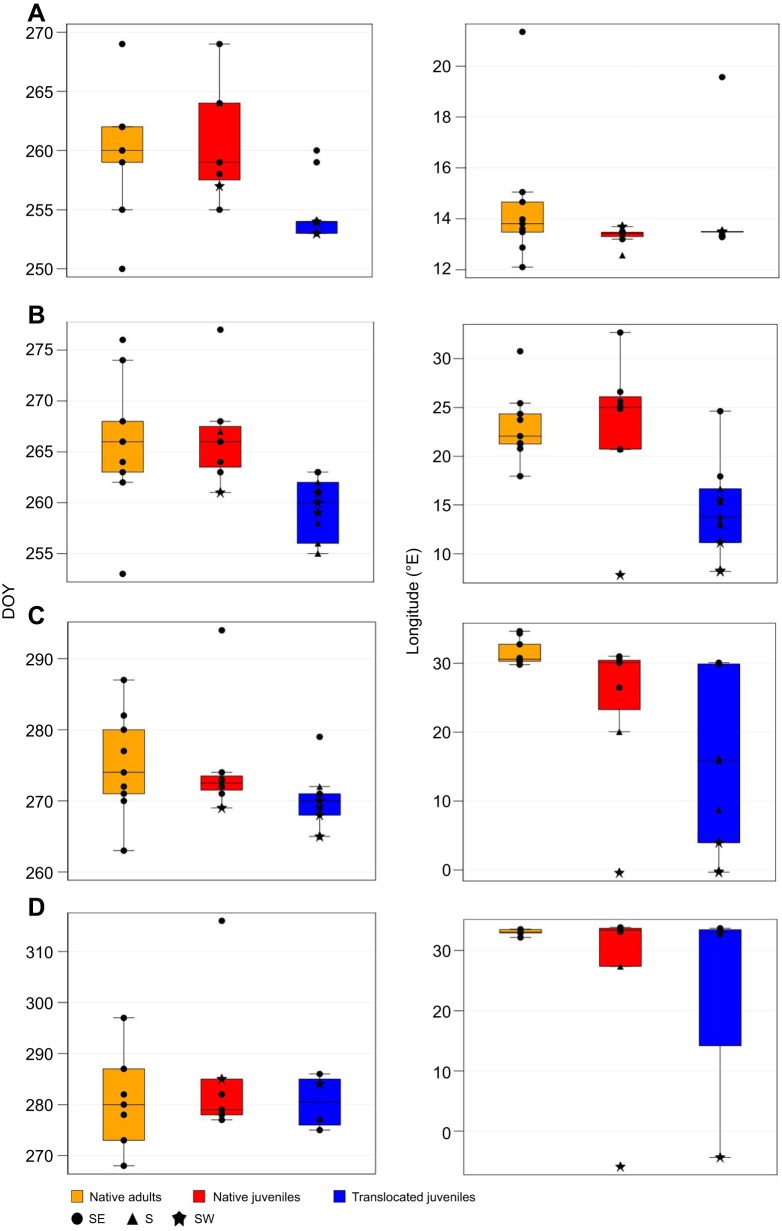
**Day of year (DOY, 1–365) and longitude at which native adults (orange), native juveniles (red) and translocated juveniles (blue) migrating from Germany departed on migration and crossed specific latitudes.** (A) Departure, (B) 50°N, (C) 40°N and (D) 30°N. Boxplots show median (bold line), interquartile range (IQR, colored box) and 1.5×IQR (whiskers). Points show individual data points according to flyway (symbol).

**Fig. 3.**
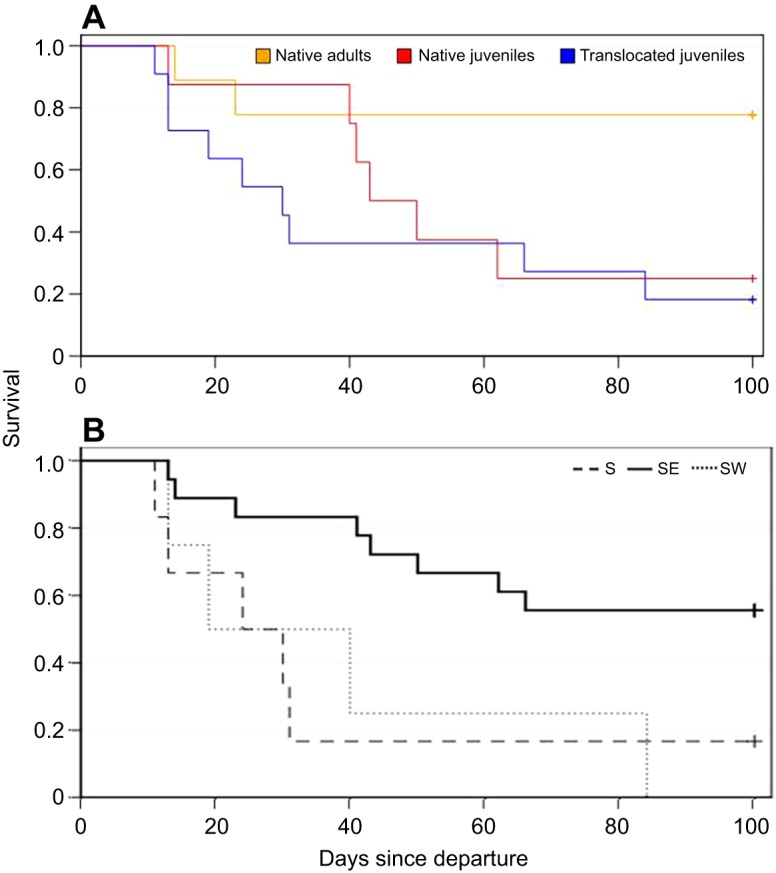
**LSE survival during the first 100 days after departing on migration.** (A) Per group; (B) per flyway.

Fig. 1 and all analyses presented in Figs S1–S6 and Tables S4 and S5 were produced using the R Language for Statistical Programming (v3.3.0, R Foundation for Statistical Computing, Vienna, Austria). We used the ‘ggplot2’ package to produce Fig. 1 (Wickham, 2009). Figs 2–3, ANOVAs and survival analyses were produced using SAS software (v9.4, SAS Institute, Cary, NC, USA).

## RESULTS

All native juveniles and adults and translocated juveniles left Germany. We excluded one adult (B23196; Table 1) from analysis in this paper because gaps in the data prohibited accurate quantification of the departure and passage timing for this bird. Translocated juvenile B94734 left the hacking station on 25 August 2009, and stopped over in northern Poland from 30 August 2009 until 17 September 2009. We considered this initial displacement as a pre-migratory move, and used 17 September 2009 as the effective departure date (Fig. 1, Table 1). One translocated juvenile, B94745, left the hacking station on 20 August 2009 (Table S1), moved to Switzerland on 28 August 2009 and starved shortly thereafter (confirmed in the field). It is unclear whether this bird was truly in ‘migration mode’ at the time it left Germany. In any case, we excluded the data for this bird from all analyses as a statistical outlier (Fig. 1, Table 1).

Table 2 summarizes the number of birds, their timing and longitude at departure and when they crossed each latitudinal threshold per group within each flyway (see Table S2 for summary statistics across all flyways per group in and Table S3 across all groups per flyway).

**Table 2.**
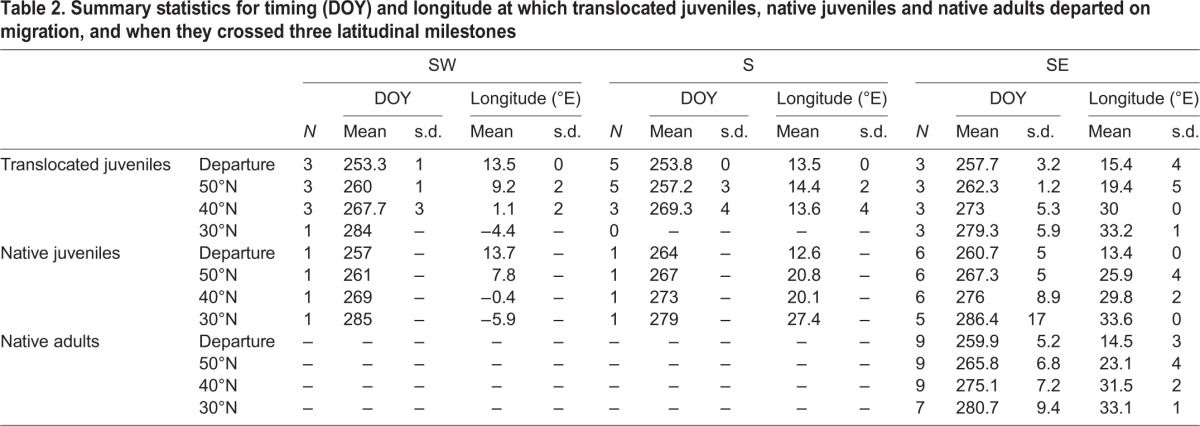
**Summary statistics for timing (DOY) and longitude at which translocated juveniles, native juveniles and native adults departed on migration, and when they crossed three latitudinal milestones**

### Route choice of translocated and native juvenile LSEs

The majority of birds using the southern/central flyway were translocated juveniles, and a three-way Fisher's exact test (Freeman and Halton, 1951) confirmed a highly significant difference in the proportion of translocated juveniles versus native birds (including native juveniles and native adults) using each of the three flyways (*P*=0.003; Fig. 1). For comparison, we show that one juvenile and one adult that were tracked from Latvia in 1993 and 1997 used the eastern detour as well (Fig. 1A), although the overland detour of the juvenile was not well detailed owing to the low resolution of the tracking data.

As all birds departed from eastern Germany, ANOVA revealed no significant difference in longitude between translocated juveniles, native juveniles and native adults at departure (Fig. 2, Table 3). The different use of flyways between groups was, of course, reflected in significant differences in the longitude at which eagles crossed latitudinal thresholds. Translocated juveniles crossed latitude 50°N significantly further west than both native juveniles (Fig. 2, Table 3) and native adults (Fig. 2, Table 3), which mostly flew eastward north of latitude 50°N. On average, native juveniles crossed each latitudinal threshold at the same longitude as native adults (Figs 1 and 2, Table 3).

**Table 3.**
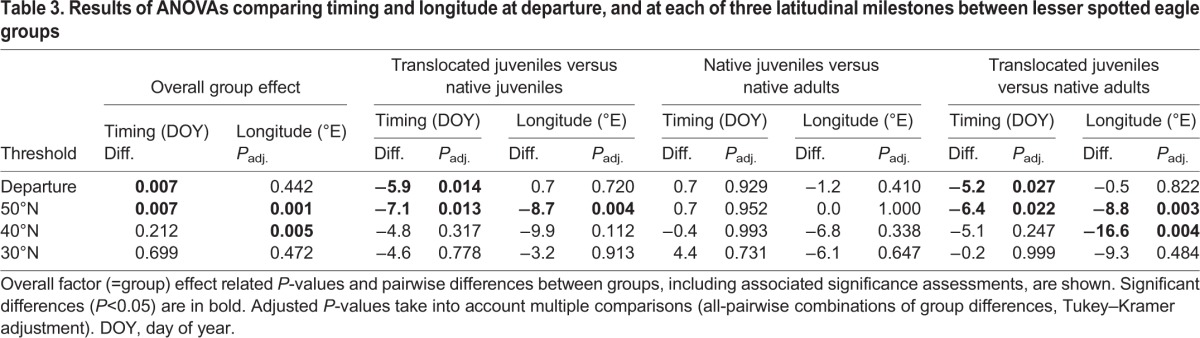
**Results of ANOVAs comparing timing and longitude at departure, and at each of three latitudinal milestones between lesser spotted eagle groups**

At 40°N, the difference in longitude between translocated juveniles and native adults, and between translocated and native juveniles remained significant, though only marginally so between the juvenile groups (Fig. 1, Table 3). At 30°N, the differences in longitude between groups were no longer significant. This increasing overlap in longitude between translocated and native juveniles with decreasing latitude was partly due to one translocated and one native bird that abruptly changed course southward while travelling over central Europe, moving away from the typical migration corridor of adult LSEs, and ending up using the central flyway (Fig. 1A). The main reason for the eventual overlap in longitudinal distributions of each group in Africa, however, was the high mortality of translocated juveniles that attempted to cross the Mediterranean (see ‘Mortality between groups and flyways’). To account for the influence of mortality, we included flyway as a covariate in the ANOVA, and this accounted for most of the differences in longitude between groups (Table 4). Only when crossing latitude 50°N were translocated juveniles flying significantly further west than native juveniles within the same flyway (Table 4).

**Table 4.**
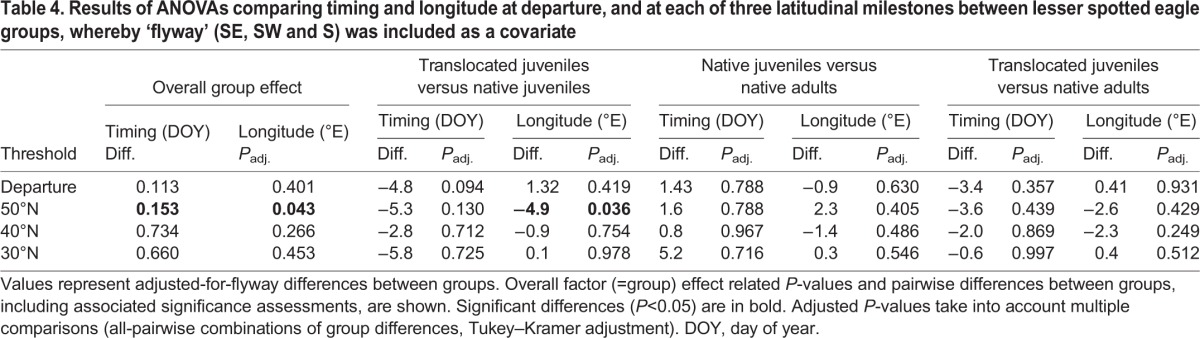
**Results of ANOVAs comparing timing and longitude at departure, and at each of three latitudinal milestones between lesser spotted eagle groups, whereby ‘flyway’ (SE, SW and S) was included as a covariate**

### Route choice and migration timing

An ANOVA revealed that translocated juveniles initiated migration, on average, 6 and 5 days in advance of native juveniles and native adults (Figs 1B and 2A, Table 3), respectively, and crossed latitude 50°N approximately 7 and 6 days before native juveniles and native adults (Table 3), respectively. The difference in the timing of migration between translocated juveniles and native juveniles and adults was not significant by the time they reached 40°N and 30°N (Table 3). The only juvenile and adult that have been tracked from Latvia departed around 17 September 1993 and 21 September 1997, respectively, comparable to the departure timing of native juveniles and native adults from Germany in 2009.

An additional ANOVA, including only flyway as an explanatory variable, revealed that the timing of the departure differed between birds using different flyways (*P*=0.033), whereby birds using the southeastern flyway departed ∼4.3 days later than those travelling south (*P*=0.049) and ∼5.5 days later than those travelling southwest (*P*=0.031). When we accounted for differences in timing between flyways by including flyway as a covariate in the ANOVA between groups, the differences in the timing at which translocated juveniles, native juveniles and native adults cross each latitudinal threshold were no longer significant (Table 4). Differences in route choice between groups were thus strongly linked to differences in the timing of each group. It is worth noting that two of three translocated eagles that used the southeastern flyway departed several days later than most other translocated eagles, with the timing of the departure being similar to that of native juveniles and adults (Table 1). Moreover, the only native juvenile to end up in the southwestern flyway departed several days earlier than all other native juveniles (Table 1), and the only native juvenile to end up in the southern flyway was one of the latest juveniles to depart, departing 2 days later than eight out of nine native adults (Table 1).

### The influence of topography and wind conditions at departure

We observed no clear relation between topography and travel direction at the start of the journey (Fig. S1). The eagles seemed to avoid high mountain ranges such as the Alps and the Carpathians in Europe (Figs S1 and S2), and migration routes were clearly shaped by the Great Rift Valley and lakes in eastern Africa (Fig. S3). Topography thus seemed to influence migration routes at local to regional scales, but it did not seem like native and translocated juveniles are funnelled along different routes by topographical features upon departure from Germany.

Generalized linear regression models revealed small but significant differences in the longitude at which birds crossed latitude 50°N owing to side wind influence at departure, in addition to the large difference between eagles of different origin (Fig. S4, Table S4). The influence of side winds was greater for juveniles than for adults (Table S5), as native adults flew east–southeast under a wide range of wind conditions (Fig. S4). When separating models between groups, we only found a significant effect of side winds on longitudinal displacement of native juveniles (Fig. S5, Table S5). However, that effect is at least partly due to the fact that departures of native juveniles were less synchronized in time than those of translocated juveniles, and therefore occurred under a much wider range of wind conditions. Moreover, under similar wind conditions, native juveniles still reached 50°N significantly further east than translocated juveniles (Fig. S4). Translocated juveniles also departed in a wide variety of directions despite wind conditions being similar on most departure dates (Figs S4 and S6). The highly synchronized departure of half of the translocated eagles on 11 September 2009 may have been triggered by a change from southerly to northerly winds (Fig. S6). However, it is unclear how selectivity for northerly winds is advantageous, as another large departure event, including two translocated juveniles, two native juveniles and three native adults on 16 and 17 September 2009, was also associated with northerly winds (Fig. S6), but with most of those birds flying to the east or southeast, instead of to the south.

### Mortality between groups and flyways

In all but one case, LSEs only reached Africa if they managed to cross the Mediterranean at Gibraltar along the southwestern flyway or migrated across the Bosphorus and Suez along the southeastern flyway (Fig. 1); native juvenile B94738 survived a long sea crossing from southern Greece to Libya (Fig. 1B, Table 1). In other words, there was a high rate of mortality in the Mediterranean region among translocated juveniles and native juveniles (Fig. 3A) along the southern and southwestern flyways (Fig. 3B). A log-rank test of survival rates confirmed that the mortality of translocated juveniles was significantly higher than that of native adults, but not different from that of native juveniles (Table 5). Only two translocated and two native individuals survived until the end of 2009 (Table 1), reaching the general wintering area in central or southern Africa that was also used by the seven surviving adults (Fig. 1B). Across groups, mortality along the southeastern flyway was significantly lower than along the southern or southwestern flyways (Table 6).

**Table 5.**
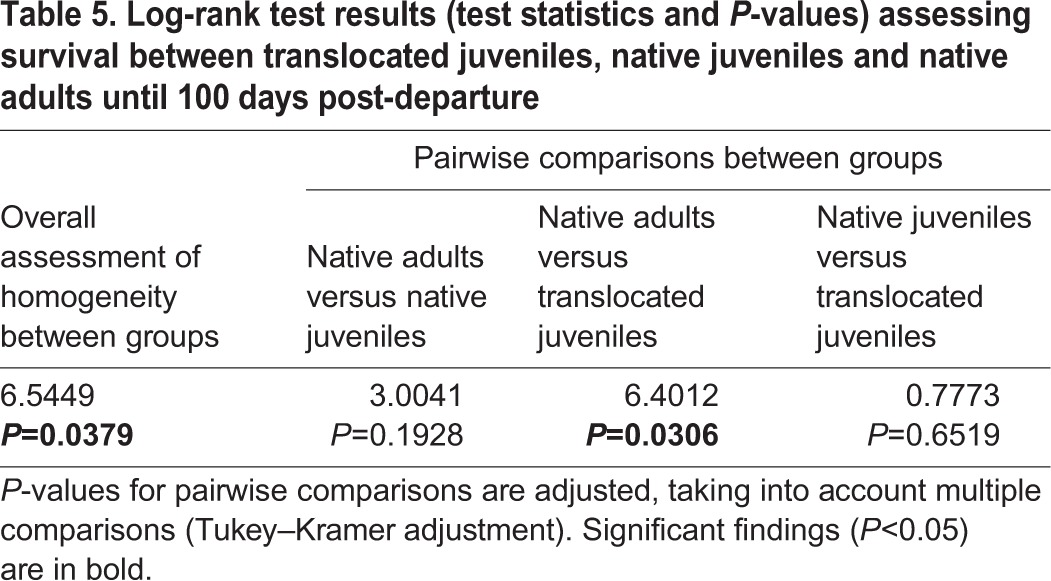
**Log-rank test results (test statistics and *P*-values) assessing survival between translocated juveniles, native juveniles and native adults until 100 days post-departure**

**Table 6.**
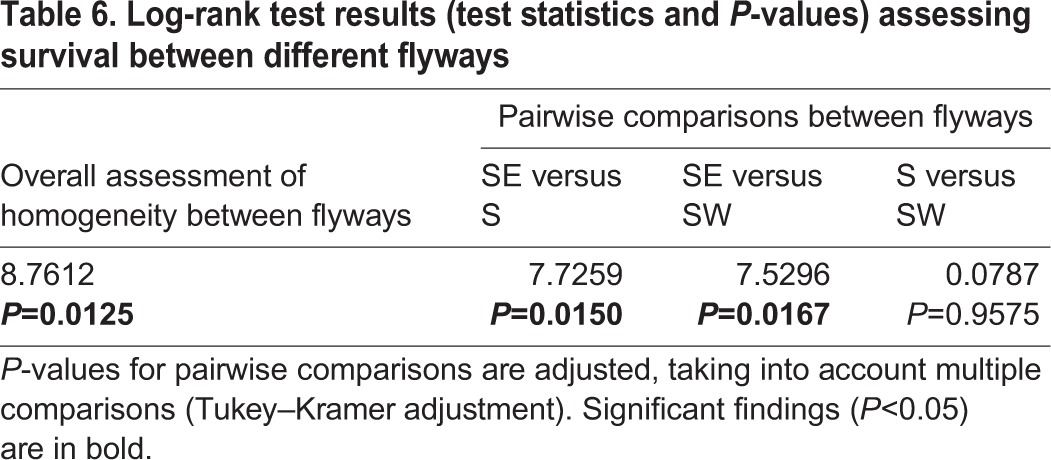
**Log-rank test results (test statistics and *P*-values) assessing survival between different flyways**

## DISCUSSION

Displacement experiments can greatly advance our understanding of migratory orientation in wild birds (Thorup et al., 2011; Guilford et al., 2011). Our findings demonstrate that the strategic use by LSEs of the eastern Mediterranean flyway and the Rift Valley has no clear genetic basis and exists on the basis of social learning. Translocated and native juvenile LSEs departing from Germany only managed to locate the southeastern flyway if they departed during the main migration period of native adults. Two of three translocated birds that made use of the southeastern flyway departed from Germany at the same time as native juveniles and adults, meaning they were more likely to encounter experienced conspecifics upon departure. In contrast, all other translocated juveniles migrated southward approximately 5 days in advance of native adults (and 6 days in advance of juveniles). Ideally, this experiment should be repeated in multiple years to better understand the influence of confounding factors (e.g. release location, weather) on departure decisions and route choice (Shamoun-Baranes et al., 2006). We found no convincing evidence, however, that the differences in timing and route choice between each group of eagles were related to the location of the hacking station relative to wild eyries, or to topography or wind conditions at departure (Walls et al., 2005). The eagles did appear to prefer northerly winds for departure, but then departed in different directions under similar wind conditions depending on their group-specific migration timing. We thus conclude that departure timing and route choice of juvenile LSEs were interlinked mainly because of the timing of social learning opportunities, and not because of changing weather conditions.

Migration timing is a rough proxy for the likelihood of juveniles encountering other LSEs, and none of the birds we tracked interacted directly with each other during migration (i.e. they migrated separately). The main exodus of LSEs occurs within a short period of time, however, as demonstrated by the similarity in departure dates of native juveniles and adults from Germany and Latvia (Fig. 1), and by the fact that 90% of all LSEs migrating through Israel pass within a period of 2 weeks, more than half passing within just a few days, and with the peak day accounting for over 20% of the total annual passage (Shirihai et al., 2000; Krumenacker, 2012). *Clanga* species are not known to migrate in family groups (Meyburg et al., 2005). Assuming that juveniles fledging in the peripheral population of Germany have relatively few opportunities to encounter passers-by, the fact that translocated, hacked juveniles departed 6 days before native juveniles and adults must have negatively affected the likelihood of translocated individuals to connect with native conspecifics or other eagles upon departure.

Contrary to our expectations, translocated juveniles departed very early in 2009, but also in a highly synchronized manner compared with native adults and juveniles, with six of the translocated juveniles departing on 11 September 2009. The access to unlimited amounts of food at the hacking stations before and after release could enable hacked juveniles to deposit fat stores faster than conspecifics that are raised in the wild (Muriel et al., 2016). The fact that these birds lived communally at the hacking station, in contrast to wild eagles, leads us to the hypothesis that social cues caused the striking synchrony in departure, whereby one or a few restless individuals could have instigated the departure of others. In the wild, eagles might also be induced to migrate when they see conspecifics passing by, which could help to explain how adults synchronize their departures. The same could be true also for fostered, translocated, Latvian juveniles. In 2007, two out of three tracked individuals migrated over the Bosphorus (Meyburg et al., 2008), where most successful LSE outward migration appears to occur. It remains unclear, however, why synchronization of departures of hacked eagles is not the same in all years (Graszynski et al., 2011), whether hacked birds depart earlier and in suboptimal directions compared with adults in all years, and how internal and external cues interact to modulate departure timing in native and translocated birds (Shamoun-Baranes et al., 2006). There remain many exciting opportunities for further research, using larger tracking datasets spanning multiple years and decades.

### Route choice in the absence of elder conspecifics

Young eagles that utilized the southwestern flyway departed early compared with juveniles and native adults using other flyways; they traveled westward, apparently irrespective of wind direction, immediately upon departure (Figs S3.1 and 3.2). The initial decision to depart westward, which is necessary for birds to end up in the southwestern flyway, has previously been suggested to have a genetic basis, an innate strategy that was more widespread when LSE were perhaps breeding farther west in Europe and when more were overwintering in West Africa (Onrubia et al., 2011). However, it is also possible that some juveniles found their way to Gibraltar by following other species of soaring migrants, or by following the Spanish coastline after entering the Iberian peninsula (Fig. 1A), as do other large soaring migrants (Mellone et al., 2011; Premuda et al., 2007). Most translocated juveniles that migrated too early to benefit from social learning departed in a southward direction, and probably had fewer opportunities to encounter experienced conspecifics or other soaring migrants compared with those that progressed towards the much busier flyways over Gibraltar and the Bosphorus. The danger of leaving early is also highlighted by one of the birds that was excluded from our analyses (B94745), which moved southward after an extremely early departure in mid-August, and died in Switzerland before any of the other translocated individuals departed on migration. Another (B94734) that departed extremely early from the hacking station initially moved eastward to Poland, where it stopped over for more than 2 weeks before departing on a concerted migration. It is unclear what motivated this bird to travel east so early, and whether that is representative of its innate migratory direction. Regardless, B94734 located the eastern flyway after leaving Poland, where there is still a large population of LSEs providing ample social learning opportunities. There was only one translocated individual that departed on 11 September 2009 that managed to locate the eastern detour. Overall, it therefore seems that the innate migratory directions of translocated birds are southward, with extremes to the west and the east.

Unfortunately, because nearly all native juveniles departed around the same time as native adults, we cannot reliably infer the innate departure directions of native juveniles. We do note one native juvenile that departed Germany very late (21 September 2009) and in an eastward direction abruptly switched course and traveled southwestward across Europe until it reached the Mediterranean coast (B94738). It is likely this bird defaulted to its innate migration program or followed another poorly navigating individual after it failed to keep up with experienced adults over Europe (Rotics et al., 2016). In either case, this bird and the individual that traveled to the southwest strongly suggest that innate departure directions of native juveniles are also highly variable and not necessarily oriented towards the eastern detour.

Other recent raptor migration studies suggest that social learning maintains strategic overland soaring migration flyways elsewhere, as shown for juvenile short-toed eagles learning the extremely detoured migration northward out of Italy in autumn before heading south through France and Spain into Africa (Agostini et al., 2016; Mellone et al., 2016). If the opportunities for juveniles to encounter experienced adults upon departure are limited in the peripheral German population, then this may limit the viability of the population and hinder westward expansion of the breeding range. Such a link between migratory behaviour and population dynamics has been demonstrated for a peripheral population of Egyptian vultures in the Balkans, where juveniles ended up traveling across and drowning in the eastern Mediterranean Sea because they failed to connect with experienced adults after departing from their natal sites (Oppel et al., 2015).

### Mortality and implications for translocation and hacking

Most juveniles that failed to locate the southeastern flyway died, probably because of starvation or persecution, after spending up to 7–10 days looking for a shorter crossing or possibly waiting for more favorable wind conditions (Gill et al., 2014; Thorup et al., 2006). LSEs are thus very reluctant to cross large stretches of open water, a characteristic seen also in juvenile short-toed eagles reared in Italy when they fail to learn the strategic detour around the Mediterranean through Gibraltar from elder conspecifics (Mellone et al., 2016). However, although short-toed eagles that fail to learn the northward route out of Italy may establish wintering sites on Sicily, LSEs are unable to survive the winter in Europe (Mellone et al., 2016). Those LSEs that became stuck at the northern Mediterranean coast probably died soon thereafter because they relied heavily on flapping flight while flying along the coast, thus exhausting their energy reserves faster than they would have by travelling along another flyway. The danger of crossing the sea is evidenced by the fact that out of three juvenile LSEs that attempted a long sea crossing, two died by drowning, similar to what happens to juvenile Egyptian vultures from the Balkans when they fail to learn an overland route from elder conspecifics (Oppel et al., 2015).

There was no significant difference in survival rates of translocated juveniles and native juveniles after 100 days, suggesting that although translocated birds from Latvia have a clear disadvantage in locating the southeastern flyway, the loss of translocated birds across the Mediterranean seems to be compensated for by other causes of mortality among native juveniles in Africa. By the end of 2009, two of 12 translocated juveniles survived compared with two of eight native juveniles. Several birds using the southwestern or southeastern flyway might have perished because of exhaustion or starvation, including those that perished in the Sahara, and several of the birds died in sub-Saharan Africa. Additional tracking efforts are needed to determine the relative contribution of natural and anthropogenic threats to mortality (1) along different flyways (Hewson et al., 2016), (2) at different stages of the annual cycle (Klaassen et al., 2014) and (3) during different life stages of this long-lived species (Meyburg and Meyburg, 2009), as well as to identify possible mechanisms of compensatory mortality between groups with different migratory behaviour.

Ultimately, the fact that several translocated birds survived their first migration suggests that the diminishing rate of population declines in LSEs in Germany in recent years is partly due to translocation and hacking efforts (Graszynski et al., 2011; Meyburg et al., 2008). All translocated juveniles were second-hatched juveniles (Abels) that would only have fledged in extremely rare instances, if not taken from their eyries (Meyburg, 1974, 1978), and even the small absolute numbers of survivors constitute a significant supplement to the relatively small Brandenburg LSE population (Böhner and Langgemach, 2004; Langgemach et al., 2010; Graszynski et al., 2011; LUNG, 2016). Although we do not know how often it happens, some translocated eagles do become breeders. The translocated female B94739, which survived almost 5 years, occupied a new territory in northeastern Poland in 2013. One color-ringed Latvian male that was translocated in 2009 (but not satellite-tracked) was first camera trapped at the hacking station when it was 2 years old, and bred successfully as an adult with an unmarked female near the hacking station at the age of five.

### Conclusions

Young LSEs must depart within the same time period as experienced adults to be able to learn the strategic route over the Bosphorus and along the southeastern flyway. In 2009, most translocated juveniles departed from the hacking station too early to follow adults and compensate for their displacement from natal areas. Therefore, they were less likely than native juveniles to learn the detour around the Mediterranean, and almost all died before reaching Africa. Hatching-year mortality, however, was relatively similar between translocated and native juveniles because many native juveniles that survived the Mediterranean ultimately died in Africa.

‘Saving Abel’ seems to be a worthwhile conservation approach, with second-hatched Latvian LSEs – and probably birds of other origin – as well as native second chicks, although translocated juveniles seem to have a harder time finding the southeastern flyway on their first autumn migration from Germany than native juveniles. These results clearly demonstrate the potential for telemetry to measure the effectiveness of costly translocation and hacking programs, to identify problems in such undertakings, and thus to facilitate evidence-based conservation of migrant birds (Muriel et al., 2016; Runge et al., 2015; Sutherland et al., 2004; Vickery et al., 2014).
